# Genomic mapping of RNA polymerase II reveals sites of co-transcriptional regulation in human cells

**DOI:** 10.1186/gb-2005-6-8-r64

**Published:** 2005-07-15

**Authors:** Alexander S Brodsky, Clifford A Meyer, Ian A Swinburne, Giles Hall, Benjamin J Keenan, Xiaole S Liu, Edward A Fox, Pamela A Silver

**Affiliations:** 1 Department of Systems Biology, Harvard Medical School and Department of Cancer Biology, Dana-Farber Cancer Institute, 44 Binney St, Boston, MA 02115, USA; 2 Department of Biostatistics and Computational Biology, Dana-Farber Cancer Institute and Harvard School of Public Health, Boston, MA 02155, USA; 3 Department of Medicine, Harvard Medical School and Department of Medical Oncology, Dana-Farber Cancer Institute, Boston, MA 02115, USA

## Abstract

**Background:**

Transcription by RNA polymerase II is regulated at many steps including initiation, promoter release, elongation and termination. Accumulation of RNA polymerase II at particular locations across genes can be indicative of sites of regulation. RNA polymerase II is thought to accumulate at the promoter and at sites of co-transcriptional alternative splicing where the rate of RNA synthesis slows.

**Results:**

To further understand transcriptional regulation at a global level, we determined the distribution of RNA polymerase II within regions of the human genome designated by the ENCODE project. Hypophosphorylated RNA polymerase II localizes almost exclusively to 5' ends of genes. On the other hand, localization of total RNA polymerase II reveals a variety of distinct landscapes across many genes with 74% of the observed enriched locations at exons. RNA polymerase II accumulates at many annotated constitutively spliced exons, but is biased for alternatively spliced exons. Finally, RNA polymerase II is also observed at locations not in gene regions.

**Conclusion:**

Localizing RNA polymerase II across many millions of base pairs in the human genome identifies novel sites of transcription and provides insights into the regulation of transcription elongation. These data indicate that RNA polymerase II accumulates most often at exons during transcription. Thus, a major factor of transcription elongation control in mammalian cells is the coordination of transcription and pre-mRNA processing to define exons.

## Abstract 2

Determination of the distribution of RNA Polymerase II within regions of the human genome identifies novel sites of transcription and suggests that a major factor of transcription elongation control in mammals is the coordination of transcription and pre-mRNA processing to define exons.

## Background

Transcriptional and post-transcriptional regulation of gene expression intersect at RNA polymerase II. The rate of polymerase II movement is altered by loading of transcription factors at the promoter, chromatin structure, pre-mRNA processing, elongation control and termination [1-3]. Thus, polymerase II accumulates at promoters as well as at different locations across a particular gene [4], but the general patterns across many different genes have yet to be explored. Numerous factors such as histones, post-translation modifying enzymes, and RNA-binding proteins regulate these processes [1,3]. One key determinant of transcription is the phosphorylation state of the carboxy-terminal domain (CTD) of polymerase II [5,6] which becomes hyperphosphorylated during transcription elongation [4,6-9]. Much of our understanding of transcription elongation comes from work in prokaryotes and yeast where most genes are intronless [1,3]. Transcription and pre-mRNA processing are coordinated, as the two processes affect the efficiency of each other [2,10]. The spatial patterns of the different phosphorylation states of polymerase II across genes remains poorly understood in mammalian systems.

## Results and discussion

To explore the range of locations where polymerase II accumulates across the genome, we performed chromatin immunoprecipitation (ChIP) from HeLa S3 cells, and profiled the purified DNA using an oligonucleotide-tiled microarray interrogating the Encyclopedia of DNA Elements (ENCODE) regions [11] covering 471 known genes. Two antibodies were used, 8WG16 and 4H8, which recognize the hypophosphorylated (PolIIa) or a phosphorylation-independent state of the CTD of polymerase II (PolII), respectively. Thus, the 4H8 antibody is recognizing the total polymerase II population. Isolated DNA was amplified using a multiple displacement amplification (MDA) strategy (see Materials and methods) [12].

**Figure 1:**
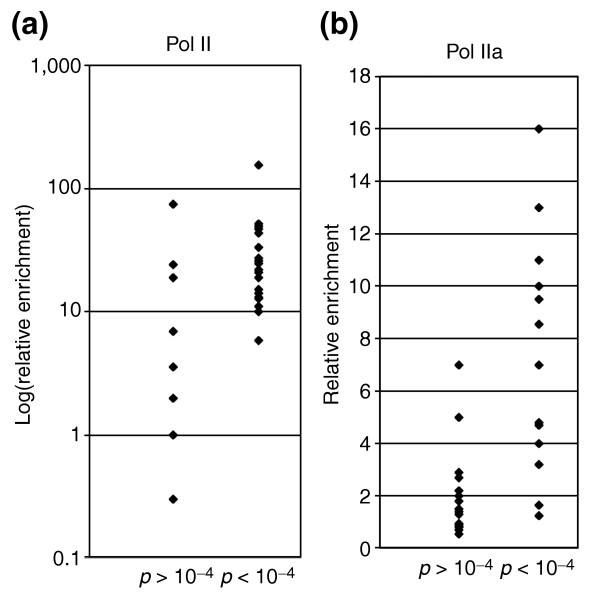
Enrichment of selected genomic regions in ChIP. **(a) **PolII ChIP; **(b) **PolIIa ChIP. Real-time PCR relative enrichment ratios for selected regions are found to be enriched more often with *p*-values below 10^-4^. These regions include both intra- and intergenic locations as listed in Additional data file 8.

To identify sites of enrichment, we used a non-parametric approach generalizing the Wilcoxon signed-rank test [13]. Signals across 1,000 nucleotides were used to determine a *p*-value for each probe. Probes were filtered for uniqueness within the bandwidth. Probes with *p*-values below 10^-4 ^were selected for further analysis because this threshold has a low false-positive rate as determined by PCR analysis (Figure 1). With these parameters, the hypophosphorylated-specific anti-PolIIa antibody reveals 102 occupied sites, whereas the phosphorylation-independent antibody shows 550 sites (Table 1).

**Figure 2:**
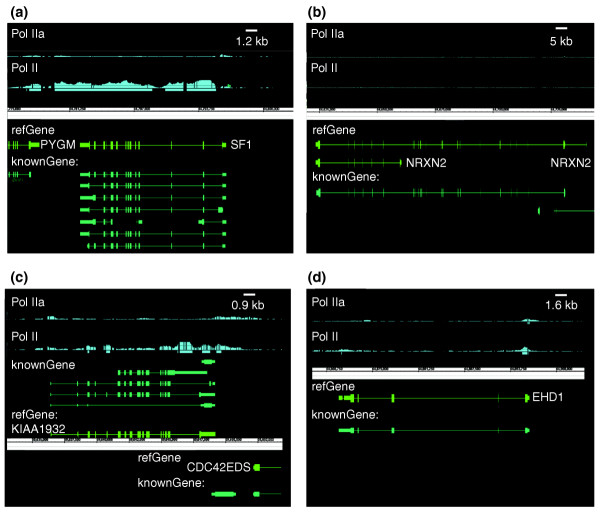
RNA polymerase II shows a variety of gene-specific enrichment patterns. Graphs plot 10log(*p*-value) mapped to chromosome position with the significant *p*-values greater than 40 indicated by the rectangle blocks below the graph. Values are plotted at every probe location. Flat lines indicate weak *p*-values and gaps indicate the absence of probes. The high density of probes across these genes suggest that the observed patterns are not due to probe bias. A scale bar is shown for each panel to reflect the different gene lengths displayed. RefSeq genes and knownGenes are annotated in green and blue, respectively, with thick bars representing exons and thin lines introns. Genes above the white bar are ordered 5' to 3', whereas those below the white bar are 3' to 5'. **(a) **On the highly expressed *SF1 *gene, PolIIa localizes to the first exon only. PolII accumulates across the gene with a distinctive pattern. **(b) **No significant signal is observed across the inactive *NRXN2 *locus which is near *SF1 *on chromosome 11. Graphs are plotted on the same scale as (a). **(c) **The moderately expressed gene *KIAA1932 *also reveals distinct accumulations across the gene. The red box highlights alternatively spliced exons. At the 3' end of the gene, some PolIIa signal is observed, probably indicative of the expression of a small gene antisense to *KIAA1932*. **(d) **Another commonly observed pattern is exemplified by the *EHD1 *gene. Both anti-polymerase antibodies recognize the first exon, but no other significant signal is observed across the gene until the 3' end.

RNA polymerase II has distinct landscapes across each gene. Figure 2 shows representative genes with polymerase enrichments. PolIIa is highly enriched at transcription initiation sites. On the other hand, PolII shows gene-specific landscapes with the strongest enrichments at exons within actively transcribed loci. Active genes reveal lower *p*-values across the gene compared with intergenic or inactive genes (compare Figure 2a and 2b), indicating a relative absence of polymerase II from the nontranscribed regions. Some smaller genes with high exon density, such as *SF1*, reveal significant polymerase signal across almost the entire locus (Figure 2a). Distinct accumulations are observed with significant *p*-values around exons for both *SF1 *and *KIAA1932*. In the *KIAA1932 *gene, PolII is enriched at a subset of constitutively and alternatively spliced exons (Figure 2c). For some genes, RNA polymerase II is enriched at relatively few locations within the gene (Figure 2d).

**Figure 3:**
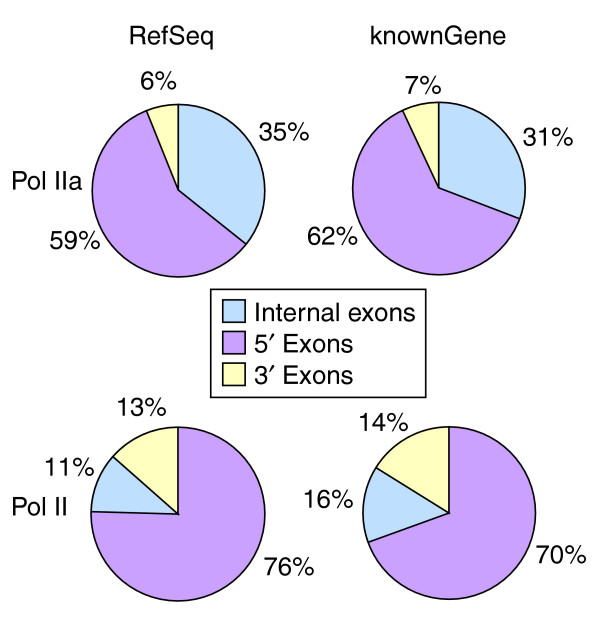
Different RNA polymerase states show distinct exon biases. Pie charts representing the percentage of exons in each category at RNA polymerase enrichment locations. These include exons from enrichment locations that include more than one exon. PolIIa is strongly biased towards transcription initiation locations. Most of the internal exons are second exons overlapping with first exons. The phosphorylation-independent antibody recognizes PolII at both transcription initiation and termination locations with a slight bias towards termination locations.

An important question is to determine if the polymerase II sites are indicative of active transcription. We addressed this in multiple ways. First, microarray expression profiling of the mRNA with Affymetrix U133 Plus 2 chips confirms that many of the RNA polymerase II-associated genes are actively expressed in HeLa cells, as seen in a plot of mRNA expression level versus *p*-value in Figure 3. Genes with significant RNA polymerase II enrichment are biased towards genes with higher mRNA levels. Figure 3 also shows that some genes have apparently high mRNA levels but no significant levels of PolII or PolIIa. This could be due to very low transcription levels but high mRNA stability. Second, we measured RNA from the same HeLa cells on the ENCODE tiled arrays. We observe that 34% of the PolII sites overlap with RNA signal (compared to approximately 8% expected at random) and 50% of the PolII locations are within 1 kb of some RNA signal (compared to 13% expected at random). Many sites where small pieces of RNA are synthesized, such as small exons, may be missed as a result of the spacing of the oligonucleotide probes and the imperfect nature of the probes. Third, many of the PolII and PolIIa sites overlap with annotated expressed sequence tags (ESTs) and mRNAs. Eighty-seven percent of the PolII-enriched and 88% of the PolIIa-enriched locations overlap with EST regions, compared to 31% and 44% expected at random, respectively. Lastly, reverse transcriptase PCR checks of *KIAA1932 *and *DKC1 *indicate that these genes are being expressed (data not shown). These data suggest that RNA polymerase II sites are biased towards regions of active transcription and that determining sites of enrichment of RNA polymerase II is an indicator of transcription.

Levels of RNA polymerase II enrichment at internal exons can vary between genes. To examine whether these patterns are influenced by expression levels, two categories were created: genes with multiple PolII enrichments at internal exons; and genes with PolII at one or zero internal exons. When compared to the mRNA levels, there is no significant difference between the two categories, suggesting that the number of PolII sites across the gene does not vary significantly with RNA levels. Genes with observable PolII enrichment at internal exons are correlated with higher mRNA levels on the expression array. This is consistent with reports proposing the use of PolII ChIP to monitor gene expression [14]. Therefore, the number of PolII sites at internal exons may reflect different levels of transcription elongation control and not just the sensitivity of the experiment.

**Figure 4:**
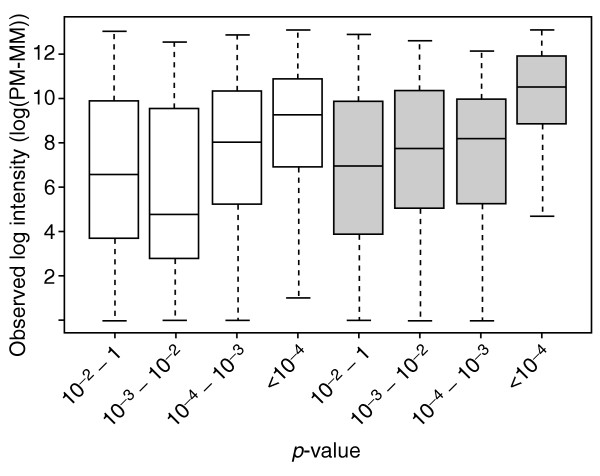
Low *p*-value PolII and PolIIa enrichments are biased towards higher mRNA levels. The plot depicts the observed intensity from Affymetrix U133 Plus 2 chips compared with different *p*-values of PolII (white) and PolIIa (gray). Some genes with no significant PolII enrichment show high levels of observed intensity.

**Figure 5:**
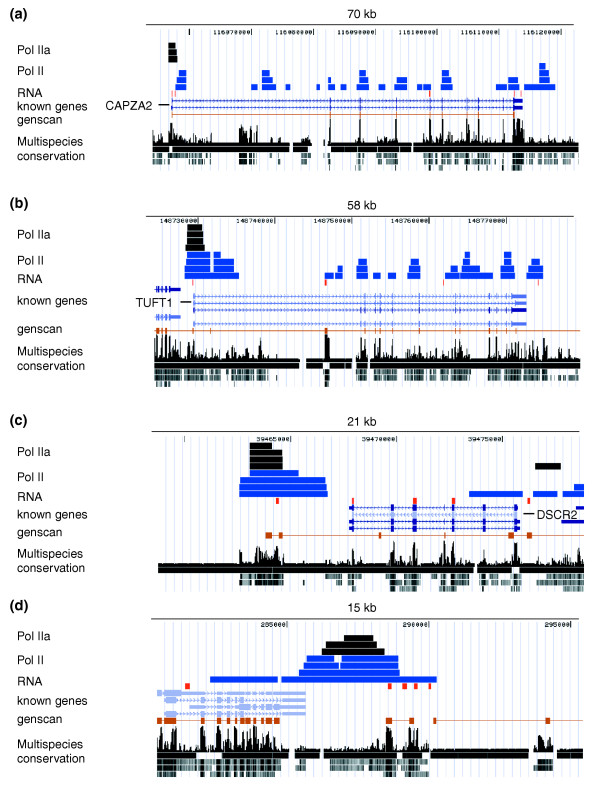
PolII enrichment is not always within annotated gene boundaries. Views are from the UCSC Genome Browser genome version HG16. PolIIa is in black and PolII is in blue with four rows for each, representing the data at different *p*-values: *p *< 10^-5^, *p *< 10^-4^, *p *< 10^-3^, and *p *< 10^-2 ^from top to bottom. RNA signal in red. **(a, b) **PolII extending beyond the 3' end of the annotated gene. **(c, d) **PolII signal in putative intergenic regions with observed RNA signal also observed in the vicinity; (d) covers chromosome 11, positions 285,000-290,000. These regions are conserved and are also near predicted genscan exons. These novel sites not in the gene regions were confirmed by PCR.

Distinct from the hypophosphorylation-specific antibody, the phosphorylation-independent antibody reveals diverse enrichment locations for PolII. In total, 74% of the identified PolII locations are near an annotated knownGene, RefSeq, or genscan exon as summarized in Table 1 (see Additional data file 2 for a list of PolII genscan exon locations). Unlike PolIIa, PolII sites are distributed between the 5' and 3' ends of genes, with a slight bias towards terminal exons over initiating exons (Figure 4). This is probably reflecting the stalling of PolII during the coupled processes of transcription termination and 3'-end processing [15]. For some genes, significant PolII signal is observed more than 1 kb past the terminal exon, which might indicate transcription of the longer pre-mRNA before 3'-end cleavage and polyadenylation [16]. Figure 5 shows two representative genes with significant PolII enrichment past the terminal exon.

Most of the hypophosphorylated PolIIa locations at internal exons also overlap a transcription initiation site, as the internal exon in question is often the second exon in the gene. Only two enrichment sites overlap with an internal exon without also being near the first exon of a transcript. One of these is at a CpG island in the *MCF2L *gene and the other may be an alternative transcription initiation site as annotated in the HG17 assembly at the beginning of the *ITGB4BP *gene. To classify the remaining sites within introns or in intergenic regions, enrichment sites were compared to other gene databases. As summarized in Table 1, four PolIIa sites are in introns, but three of these are within resolution of annotated or predicted exons, leaving only one location not overlapping an exon of some kind. There are 28 hypophosphorylated polymerase sites not in a RefSeq gene region. After following a similar filtering approach, only 14 sites remain that are not near a putative exon. Thus, only 14% of PolIIa-enriched locations do not overlap with a known exon or actively transcribed region. Additional data file 2 lists PolIIa sites at predicted exons that are probably newly identified transcription initiation locations in HeLa cells. Figure 5 shows two examples of PolII and RNA signal at new sites of transcription. From the pattern of enrichments it is probable that many of these predicted exons are real and are transcription initiation locations, given the observed strong bias of the 8WG16 antibody for transcription initiation locations in well annotated genes.

To determine the generality of these observations, all RNA polymerase II occupancy sites were compared with the known genes and RefSeq databases, version HG16. PolIIa is highly enriched for the first exons around transcription initiation sites (Figure 4) representing 77 of 551 known genes in HG16 on the array (see Additional data file 1 for the entire lists).

Elongation control is a common transcriptional regulation mechanism believed to affect a wide range of functional gene classes [1]. In particular, RNA polymerase II pausing has been proposed to be associated with alternative splicing, [2]. To determine if there is a bias for alternative exons, we counted all the annotated alternatively spliced exons in the knownGene database and determined the distribution of PolII enrichment locations on them. PolII is enriched at 57% of the annotated alternatively spliced exons of the active genes compared to 37% of annotated actively transcribed constitutively expressed exons. We also examined the distribution of all PolII *p*-values on different types of exons. Each exon was mapped to the smallest *p*-value ChIP-enriched site that overlaps the exon. The cassette exons are found to be more significantly associated with smaller *p*-values compared to constitutively expressed exons according to the two-sample Kolmogorov-Smirnov test with a two sided *p*-value of less than 0.0035.

One attractive hypothesis is that sites of exon enrichment may reflect weaker splice sites where PolII stalls during splice site recognition. Using two different empirical methods to estimate splice site strength, no significant differences are observed between the exons overlapping PolII and those that do not [17,18]. Alternatively, some of the annotated constitutively expressed exons may actually be subject to alternative splicing decisions. Kampa *et al*. suggest that the levels of alternative splicing are much higher than commonly believed and annotated in the human genome from their examination of expression on tiled arrays [19]. Consistent with these findings, RNA polymerase II sites may be predicting which exons are being co-transcriptionally alternatively spliced.

To determine if there is any pattern for the 120 PolII enrichment sites that are in RefSeq introns, we compared these sites to knownGene, genscan, geneid, and sgpGene databases and find 31 within resolution of putative exons. Of the remaining 89, 57 are in genes with PolII enrichment sites that also overlap exons, suggesting that they are actively transcribed genes. No clear intronic positional bias is observed.

In conclusion, we have identified new sites of RNA polymerase II accumulation across hundreds of genes in mammalian cells. The large majority of polymerase II-enriched locations are at actively transcribed exons with a bias towards annotated alternatively spliced exons. Many of the PolII sites at annotated constitutively expressed exons may be sites of alternative splicing. Whatever the eventual splicing decision, these observations suggest that events around exons slow transcription elongation. A recent study suggests that even general splicing factors may slow elongation [20]. Stalling of RNA polymerase II near exons may function to slow RNA synthesis in order to wait for the competition of myriad splicing signals to be resolved in order to define the exon [21,22]. These ChIP data identify where these states of RNA polymerase II are localizing across the ENCODE regions.

Across genes, these data are consistent with the hypothesis of transcriptional pausing at particular locations. Alternatively, it is possible that RNA polymerase II is rearranging during transcription such that the epitope is only accessible around exons. Thus, the conformation of polymerase II may be changing and not the transcription rate. Nonetheless, it is interesting that the majority of observable elongating polymerase II accumulates around exons, suggesting that a major feature of transcription elongation control is coupling to pre-mRNA processing.

These observations differ from those observed in intronless genes typically found in prokaryotes and yeast where a more uniform PolII enrichment is observed across genes [16]. What appears to be conserved is PolII accumulation in coding regions compared to intronic regions. These data highlight the complexity and gene-specific nature of transcription regulation not only at transcription initiation and termination locations but at specific exons. Together, these observations suggest that a major feature of transcription elongation control in mammalian cells is exon definition. Thus, these data provide new insights into the coordination of transcription and pre-mRNA processing in mammalian cells.

## Materials and methods

### Chromatin immunoprecipitation and DNA amplification

Chromatin immunoprecipitations (ChIP) were performed as described with the following modifications [23]. HeLa S3 cells were first crosslinked with dimethyl adipimidate (DMA) (Pierce) for 10 min, washed with PBS and then crosslinked with formaldehyde for 10 min. Cells were collected, lysed, and chromatin was sheared by sonication to an average length of 1 kb as determined after RNase treatment of the samples on an agarose gel. Chromatin was prepared from four independently grown batches of cells and pooled to generate three replicate immunoprecipitations (IP) and six input samples. Briefly, 8WG16 (Covance) and 4H8 (AbCam) antibodies were incubated with a 50:50 mix of Dynal protein A/G beads for more than 16 h at 4°C in PBS with 5 mg/ml BSA. After washing in PBS, beads with bound antibody were incubated with chromatin from approximately 2 × 10^7 ^cells for more than 16 h at 4°C. Beads were washed eight times with RIPA buffer (50 mM HEPES pH 7.6, 1 mM EDTA, 0.7% DOC, 1% IGEPAL, 0.5 M LiCl) before DNA was eluted at 65°C in TE/1% SDS. Crosslinks were reversed by incubating at 65°C for more than 12 h followed by proteinase K treatment, phenol extraction and RNase treatment. Isolated DNA was then amplified isothermally using random nonamer primers and Klenow polymerase (Invitrogen) for more than 4 h, yielding approximately 2 μg of DNA per IP. DNA was prepared and hybridized on Affymetrix ENCODE oligonucleotide tiled arrays using the fragmentation, hybridization, staining and scanning procedure described by Kennedy *et al*. [24]. Affymetrix ENCODE microarrays have interrogating 25mer oligonucleotide probes tiled every 20 bp on average. A sample of chromatin was set aside before IP and used to represent the input DNA.

### Tiled array analysis

Quantile normalization was used to make the distribution of probe intensities the same for all arrays [25]. In the case of the Affymetrix GTRANS software quantile normalization is used within treatment and control replicate sets. Non-parametric methods based on ranks were used to identify ChIP-enriched regions. These methods make mild assumptions about the data distributions and are insensitive to outlying observations. A *p*-value was calculated for every assay probe on the array. The set of probes used in the calculation of this *p*-value was defined by a bandwidth parameter *b*. All probes centered on the chromosome at positions less than *b *bases 5' or 3' of the given probe position are included in this set.

The Wilcoxon rank sum test [26], also known as the Mann-Whitney U test, is the basis of the *p*-value statistic computed by the Affymetrix GTRANS software. The control and treatment observation sets are, respectively, the sets of normalized control and normalized treatment intensities from all replicates and all probes within the bandwidth. The null hypothesis is that the treatment set mean is no larger than that of the control set.

To take into account probe-to-probe variability we used a generalization of the Wilcoxon signed-rank test for blocked data. All input and IP normalized, sign(PM-MM)max(1,|PM-MM|) intensities (where PM are perfect match and MM are mismatched probes) interrogating the same chromosomal location were assigned to the same block. Aligned observations were derived by subtracting the median normalized intensity for a given block from each observation in that block. All aligned observations within the bandwidth were ranked. A statistic W was defined as the sum of the ranks of the aligned IP observations. A *p*-value was derived from W, based on the joint null distribution of the aligned input and IP ranks. The analyses depend on the assumption that probes are independent. Probes were mapped to the genomic coordinates to ensure that no probe mapped to more than one location in any 1,000-bp window and that no two probes map to the same genomic location.

### RNA arrays

RNA samples were isolated from HeLa S3 cells and purified with trizol (Invitrogen) and RNeasy (Qiagen). RNA was amplified and hybridized to Affymetrix U133 Plus 2 arrays using standard methods. Three biological replicates were quantile normalized. Gene expression was indicated by the median of PM-MM values over all probes. The hypothesis of difference in gene expression between groups of genes, based on median PM-MM, was tested using the Wilcoxon rank sum statistic. For hybridization to the ENCODE tiled array, RNA was similarly isolated and double-stranded cDNA was generated using Invitrogen Superscript cDNA synthesis kit. cDNA (1-1.5 μg) was hybridized to the tiled array. Three biological replicates were performed for each RNA array.

### Genomic annotation

Sites were determined to be near a genomic annotation if they were within the apparent 1,000 bp resolution. Sites shorter than 1,000 bp were scaled in size to include 1,000 bp around the center of the site. Sites that were longer than 1,000 bp used the data-determined length for their resolution size. Databases were downloaded from the University of California at Santa Cruz (UCSC) Golden Path Genome Browser and loaded into a local MySQL database. Exons were compared and classified as one or more of the following: start, terminal, alternatively spliced, constitutive or cassette. Because the arrays were designed using the HG15 assembly, the data were compared to this version of the human genome unless otherwise noted. The active gene list was defined as those with PolIIa at the first exon of the gene.

### Real-time PCR

PCR primer pairs were designed to amplify 100-bp fragments from selected genomic regions (see Additional data file 8). Each real-time PCR reaction contained 50 nM primers, approximately 1 ng DNA and 1 × ABI SYBR PCR reaction mix. A fluorescence value proportional to the initial quantity of target DNA was calculated by a log-linear regression analysis for each quadruplicate amplification curve [27]. We normalized this value to an input chromatin sample, then normalized this ratio to a reference gene, *PAPT*, which is not expressed in HeLa cells, to calculate a relative enrichment value for the target ((Target_IP_)/(Target_Inp_))/((PAPT_IP_)/(PAPT_Input_)).

### Data availability

All data is present at Gene Expression Omnibus (GEO) at accession number GSE2735.

## Additional data files

The following additional data are available with the online version of this paper. Additional data file 1 is a table listing PolIIa annotated to refGene. Additional data file 2 is a table listing PolIIa annotated to known genes. Additional data file 3 is a table listing PolIIa annotated to RefSeq. Additional data file 4 is a table listing PolII annotated to known genes. Additional data file 5 is a table listing PolII annotated to genscan exons. Additional data file 6 is a table listing knownGene and RefSeq populations on the ENCODE array. Additional data file 7 is a table listing the PolIIa-defined active gene list. Additional data file 8 is the PCR primer list and annotation.

## Supplementary Material

Additional File 1 A table listing PolIIa annotated to refGene. Click here for file

Additional File 2 A table listing PolIIa annotated to known genes. Click here for file

Additional File 3 A table listing PolIIa annotated to RefSeq. Click here for file

Additional File 4 A table listing PolII annotated to known genes. Click here for file

Additional File 5 A table listing PolII annotated to genscan exons. Click here for file

Additional File 6 A table listing knownGene and RefSeq populations on the ENCODE array. Click here for file

Additional File 7 A table listing the PolIIa-defined active gene list. Click here for file

Additional File 8 The PCR primer list and annotation. Click here for file

## Figures and Tables

**Table 1:** Summary of RNA polymerase II locations

Sites		Pol IIa	Pol II
Total sites		102	550
RefSeq total exons		70	289
RefSeq first exons		63	75
RefSeq terminal exons		2	91
RefSeq internal exons		5	123
			
RefSeq introns		4	120
	knownGene exon	0	5
	genscan exon	1	23
	geneid or sgpGene	0	3
	Active gene introns	2	57
	Inactive introns	1	32
			
No RefSeq overlap		28	141
	knownGene total exons	5	38
	knownGene first exon	5	13
	knownGene terminal exon	0	4
	knownGene internal exon	0	21
	No RefSeq or knownGene	23	90
	genscan exons	7	43
	geneid or sgpGene	2	6

The order indicates the flowchart of filtering through the different databases. Enrichment sites were first compared to the RefSeq database. Sites that are not near exons were then divided into two categories: locations that are in RefSeq introns; and locations that are not in a RefSeq gene. The latter are then compared with knownGene and predicted gene databases. For both RNA polymerase II phosphorylation states, the large majority of sites are near an exon.
